# Event and Comment

**Published:** 1921-08

**Authors:** 


### DENTAL REGISTER

THE MAGAZINE OF DENTISTRY

Vol. LXXV AUGUST, 1921 No. 8

### EVENT AND COMMENT

**What Further is Demanded of Dentistry in View of Our Present Standards and Practices?**

We are printing in this issue three papers which we believe ought to be carefully read and studied, because they furnish texts for consideration of probably the most important matter the dental profession will have to work out in the near future. Our health problem is and has always been a most complicated one, and it seems, so far as dentistry is concerned, none the less a more vital problem at this time than ever before.

We do not care to discuss this matter at length at this time, for it would require more wisdom and skill than we command. The views expressed in the papers referred to, are well worth while, being carefully read and even studied at great length, because most opportune.

We present these papers, not in the order of their importance particularly, but as an orderly presentation of events with the idea, giving the details which called forth the incidents which prompted the general subject for consideration, and its timeliness. We would rather not discuss the particular items now, as each paper has done this with sufficient detail and excellence.

Many items in the first paper give us ample opportunity for critical review, and we could not agree with some of the essayist's logic. Our purpose now is to keep in mind the general theme and so see if we can not see a crystallizing of a sentiment of unity and co operation.

We hope, therefore, the general subject only may be kept in mind in the study of these papers. As we see it, these papers are inclined to consider something other than technical matters or traditions of practice. What we need more than these details of transient expedients are substantial findings of scientists and workers who will give us fundamental data upon which to construct rules of abiding practice, because founded on known fundamentals.

We all hope we shall, in the near future, have more fundamentals to work with than now, but this need not deter anyone from using the best theoretical knowledge attainable until we shall have found a more enduring basis for practice, else our field of art will be limited indeed.

What we have in mind in presenting this symposium is to excite a stimulus to utilize what seems to us most timely in these papers on these allied subjects, and to cause them to re-act profitably upon our readers.

Dr. Talbot thinks our educational system is faulty, and it should either be revamped or be broadly enlarged instead of making it technically more efficient. Both may be probable, but we know from experience that such a proposition will not meet with general dental consent. Proof positive and patient and tactful leadership only will convince the profession.

Dr. Moorehead intimates that early prophylaxis or care of children's teeth; safe operation and surgical interference, when required, are matters of approved treatments and can be utilized in the majority of cases; but he also says we must have a better knowledge of scientific medical facts than is generally obtained by dental practitioners.

Professor Colyer has given us a most comprehensive outline of the physiologic and pathologic bearings on dental practice, especially on the related systemic influences. His article is rather extended, but it is exceptionally well worth careful consideration. It is the kind of information that will make technical dentists think of the part the practitioner must absorb and put into co-ordination with the physician and the dentist.

From the nature of the case we have absorbed our art traditionally and we have found out that we can not be dental artisans and pseudo medical practitioners. Since we can not be dentists without knowing something of the healing art, it seems rational that we should learn all the healing art of medicine that science can give to us. Dr. Talbot suggests it may be possible for dentists to learn more bacteriology and pathology than we used to think necessary; if so, we can do this, and we will do it when we know the reasons for so doing.

